# News Items

**Published:** 1872-01-15

**Authors:** 


### News Items.

The issue of *The Clinic* for December 23d, 1871, might be appropriately styled a "*small pox number*," it being exclusively devoted to the consideration of that disease. Its contents comprises, "A History of Small-pox, compiled from various sources," original articles on the treatment of Small-pox at Rahs Hill Hospital, by Chas. P. Judkins, M. D., "Hemorrhagic Small-pox, by J. L. Cleveland, M. D.," and "Eye Complications in Small-pox, by W. W. Seely, M. D." Also, miscellaneous scientific notes, clinical memoranda, etc., all relating to Small-pox and vaccination.

*The Clinic* gives the number of deaths from Small-pox in Cincinnati during the week preceeding December 26th, 1871, as sixty-two, the whole number of deaths for the same time being one hundred and thirty-one.

Medical Department of Harvard University.—The number of students in all the departments of Harvard University is not so large as last year by 210—a falling off of 105 medical students.

The important change in the latter, in the plan of study and the requisites for a medical degree, probably accounts for the deficiency in numbers.—*Ibid.*

Chlorate of Potassa in Dysentery.—Dr. A. B. Isham, of Cincinnati, Ohio (*The Clinic*), says that in cases of chronic purulent dysentery, which has resisted the usual mode of treatment, he has resorted to the use of chlorate of potassa with success.

M. Nelaton.—It is rumored that this eminent French surgeon is to settle in London.

The Third Volume of the late Sir James Y. Simpson's works, comprising Diseases of Women, and completing the series, will appear early in January.

A Lengthy Skeleton.—Capt. Burton, the English explorer, is bringing home from Palmyra, besides a rare collection of skulls, a human skeleton eleven feet in length.

